# Balloon Cell Melanoma: Presentation of Four Cases with a Comprehensive Review of the Literature

**DOI:** 10.3390/dermatopathology9020013

**Published:** 2022-03-28

**Authors:** Gerardo Cazzato, Eliano Cascardi, Anna Colagrande, Antonietta Cimmino, Giuseppe Ingravallo, Lucia Lospalluti, Paolo Romita, Aurora Demarco, Francesca Arezzo, Vera Loizzi, Miriam Dellino, Irma Trilli, Emilio Bellitti, Paola Parente, Teresa Lettini, Caterina Foti, Gennaro Cormio, Eugenio Maiorano, Leonardo Resta

**Affiliations:** 1 Section of Pathology, Department of Emergency and Organ Transplantation (DETO), University of Bari “Aldo Moro”, 70124 Bari, Italy; anna.colagrande@gmail.com (A.C.); giuseppe.ingravallo@uniba.it (G.I.); emilio.bellitti@gmail.com (E.B.); teresa.lettini@uniba.it (T.L.); eugenio.maiorano@uniba.it (E.M.); leonardo.resta@uniba.it (L.R.); 2 Section of Pathology, Department of Medical Sciences, University of Turin, 10121 Turin, Italy; eliano20@hotmail.it; 3 Section of Dermatology, Department of Biomedical Science and Oncology (DIMO), University of Bari “Aldo Moro”, 70124 Bari, Italy; l.lospalluti@gmail.com (L.L.); paolo.romita@uniba.it (P.R.); aurorademarco94@gmail.com (A.D.); caterina.foti@uniba.it (C.F.); 4 Section of Gynecology and Obstetrics, Department of Biomedical Sciences and Human Oncology (DIMO), University of Bari “Aldo Moro”, 70124 Bari, Italy; francesca.arezzo@uniba.it (F.A.); vera.loizzi@uniba.it (V.L.); miriamdellino@hotmail.it (M.D.); gennaro.cormio@uniba.it (G.C.); 5 Clinic of Obstetrics and Gynecology, “San Paolo” Hospital, ASL Bari, 70124 Bari, Italy; 6 Odontomatostologic Clinic, Department of Innovative Technologies in Medicine and Dentistry, University of Chieti “G. D’Annunzio”, 66100 Chieti, Italy; irmatrilli@hotmail.com; 7 Pathology Unit, Fondazione IRCCS Casa Sollievo della Sofferenza, 71013 San Giovanni Rotondo, Italy; paolaparente77@gmail.com

**Keywords:** malignant melanoma, balloon cell, differential diagnosis, dermatopathology

## Abstract

Background: balloon cell melanoma represents less than 1% of all histological forms of malignant melanoma and represents a diagnostic challenge for the dermatopathologist. Methods: in this paper we present our cases of BCM found in our daily practice from 1 January 2008 to 31 December 2021, and we conduct a review of the literature relating to this entity in the period from the first description, 1970, to early 2022. Results: four cases of melanoma balloon cell have been extrapolated from our electronic database, while in the review of the literature we have identified 115 cases of patients with primary and/or metastatic BCM. Conclusions: we believe that future studies with numerous case series are essential not only to increase the knowledge of the pathophysiology of this neoplasm but also to correctly evaluate the response of BCM patients to new oncological therapies.

## 1. Introduction

Malignant melanoma poses an ongoing challenge for health systems across the globe, and incidence and prevalence rates continue to rise, making prevention a crucial issue [1]. It is known that the histological diagnosis has a fundamental significance in the correct nosographic classification, which supports decision making and planning of the different therapies [2]. However, the diagnosis is not always easy, and every day the dermatopathologist has to deal with complex pictures that require integration with immunohistochemical and molecular data. Furthermore, this neoplasm can arise at the level of other parts of the body, such as mucous sites including the oral cavity [3], the vagina [4] or intestine [5]. In this context, balloon cell melanoma (BCM), is a fairly rare, bizarre entity that can sometimes manifest not only in a context of melanoma metastases but also as a primary lesion [6]. Over time, different explanations have been proposed to justify the morphological changes, but ultimately, the best accepted view (also thanks to electron microscopy studies and acquisitions) is that an overproduction of swollen and defective melanosomes is at the origin of this morphotype [7]. In this paper, we present four cases of balloon cell melanoma, discuss their main differential diagnoses and perform an extensive review of the current literature in order to trace the state of the art and future prospects.

## 2. Materials and Methods

To carry out this work, the historical archive of our laboratory was consulted from 1 January 2008 to 31 December 2021, applying the term “Balloon Cell” for the search, so that only cases of malignant melanoma were extrapolated. Sections staining with Hematoxylin/Eosin (EE) and blocks were retrieved and re-analyzed by two pathologists with expertise in skin pathology (G.C. and A.C.). In the event that there was no agreement, a third dermatopathologist (C.A.) was included in the discussion. Clinical information was retrieved from fellow dermatologists and plastic surgeons, and, when not available, the patient or family members were contacted directly. In addition, a systematic review was elaborated following the Preferred Reporting Items for Systematic Reviews and Meta-Analyses (PRISMA) guidelines. A databases search of PubMed, Web of Science (WoS) and Scopus was performed for the period 1970–2021 using the following terms: balloon cell melanoma and melanoma with balloon cell in combination with each of the following: dermatopathology, skin. Only articles in English were selected. The last search was run on 31 December 2021. Eligible articles were assessed according to the Oxford Centre for Evidence-Based Medicine 2011 guidelines [8]. Review articles, meta-analyses, observational studies, case reports, survey snapshot studies, letters to the editor and comments to the letters were all included. Other potentially relevant articles were identified by manually checking the references of the included literature. An independent extraction of articles was performed by two investigators according to the inclusion criteria. Disagreement was resolved by discussion between the two review authors.

## 3. Results

Four cases of melanoma balloon cell have been extrapolated from our electronic database, the clinical-pathological characteristics of which are reported in Table 1.

**Figure 1:**
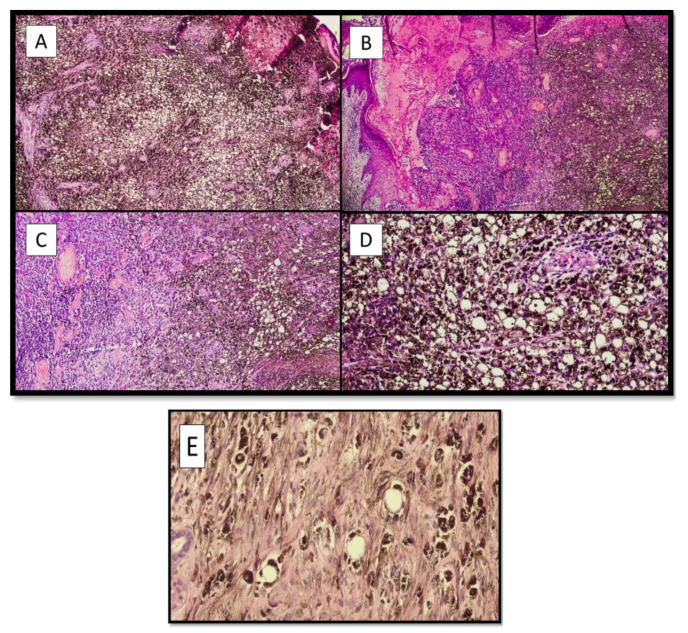
(**A**) Photomicrograph of the first case (F, 76 years old), showing the pseudolipoblastic/balloon cell aspects of the melanocytes, characterized by a swollen histological appearance and the disintegration of disordered and abundant melanic pigment (Hematoxylin-Eosin, Original Magnification 4×). (**B**) Histological micrograph of patient number two (M, 75), showing two different neoplastic parts: on the right, the more properly “balloon cell” part, while in the center and on the left, there is a part with spindle cells of malignant melanoma. Additionally, in this case, there was an abundant and irregular presence of melanic pigment (Hematoxylin-Eosin, Original Magnification 4×). (**C**) Histological preparation of sections from the third patient (F, 36 years old) showed very similar morphological characteristics to those in case number two (Hematoxylin-Eosin, Original Magnification: 10×). (**D**) Balloon cell melanoma photomicrograph of the lesion in patient number four (M, 51 years old). Note the balloon-shaped appearance of melanocytes with histological characteristics that sometimes resemble pseudolipoblasts (Hematoxylin-Eosin, Original Magnification: 20×). (**E**) Histological micrograph showing the cytological detail of melanocytes with balloon cell characteristics of the cytoplasm (Hematoxylin-Eosin, Original Magnification: 40×).

Records of two male (50.0%) and two female patients (50.0%) were retrieved, with balloon cell melanoma localizations in four different body districts. In three of the four cases (75.0%) the clinical suspicion was malignant melanoma. Microscopically, all the lesions had the same characteristics, consisting of more than 50% of “balloon-shaped” melanocytes. (Figure 1A–C). These cells featured an abundant and finely vacuolized cytoplasm and hyperchromatic nuclei, generally located in the periphery of the cell, but not pycnotic (Figure 1D). Very rare mitoses were observed, and melanin was quantitatively reduced within the cell, with a “disordered” dispersion within the lesion itself and in the numerous melanophages (Figure 1D). Architecturally, in all four cases, the cells were organized in large pale masses that replaced the dermis and seemed to thin the epidermis (Figure 1B). These large solid sheets of “ballooniform” melanocytes were divided into irregular aggregates by thin collagenous septa. There were no clear signs of activity at the dermo-epidermal junction and/or pagetoid spreading. In the second case (Figure 1B), a component of “spindle cell” melanoma could be observed.

**Figure 2:**
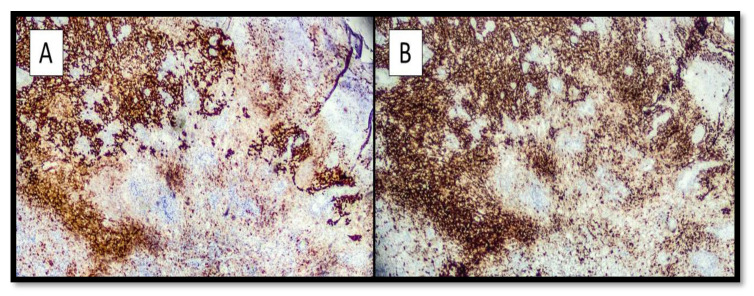
(**A**) Immunohistochemical preparation with S-100 protein antibody: note the intense positivity of the marker at the level of the melanoma component with spindle cells and a more tenuous positivity at the level of the balloon cell component. (Immunohistochemistry, Original magnification: 10×). (**B**) Photomicrograph showing immunostaining with anti-Melan-A antibody: note that the positivity of staining is almost entirely comparable to the previous one. (Immunohistochemistry anti-Melan-A, Original Magnification: 10×).

Immunohistochemically, all four cases expressed S-100 protein and Melan-A (Figure 2A,B), as well as positivity for HMB-45 and SOX-10.

**Figure 3:**
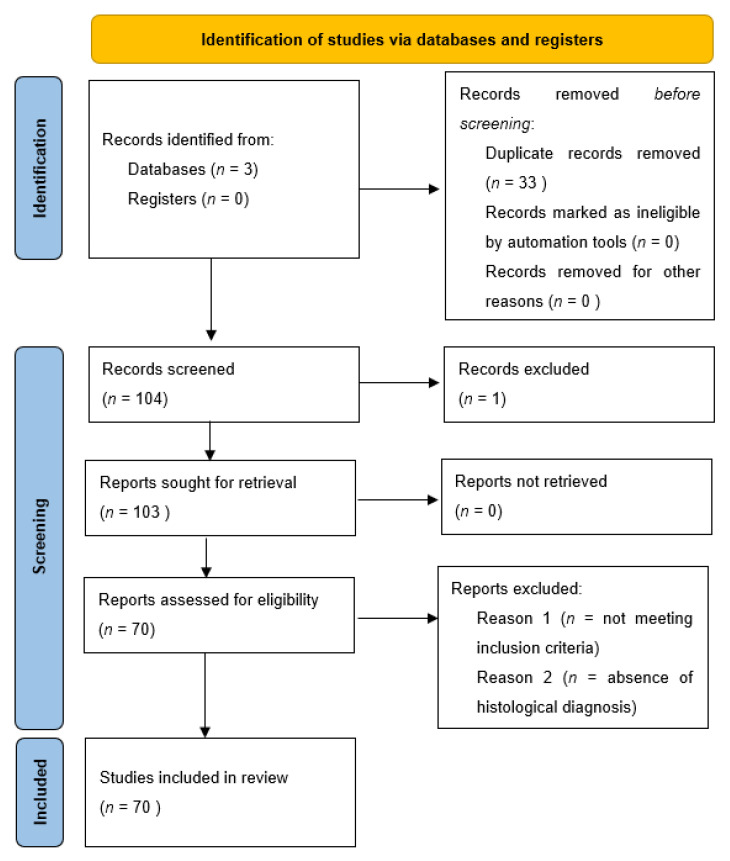
PRISMA 2020 Flow chart utilized for this systematic review related to balloon cell melanoma.

In the review of the literature, a total of 137 records was initially identified, of which 33 were duplicates. After screening for eligibility and inclusion criteria, 70 publications were ultimately included (Figure 3). The authors and clinical/pathological characteristics are summarized in Table 2. Most of the publications were case reports (*n* = 51), followed by reviews (*n* = 10), case series (*n* = 6) and editorials (*n* = 3). All studies included were rated as evidence level 4 or 5 for clinical research, as detailed in the Oxford Centre for Evidence-Based Medicine 2011 guidelines [8]. In total, 115 patients with primitive or metastatic balloon cell melanoma were described.

Of these 115 patients, 36 (31.3%) had a primary lesion starting in the back (1 case starting in the left shoulder blade); 20 (17.4%) a lesion starting in the extremities (17 cases in the upper limbs and 3 cases in the lower limbs); 11 patients (9.6%) had a primary head/neck lesion; 9 patients (7.8%) had primary BCM of the choroid or ciliary body, while 2 patients (1.7%) had BCM originating in the conjunctiva. Metasases were present in 15 patients (13.0%) at the time of observation, while in 9 cases (7.8%), the site of the first melanoma was unknown. Finally, there were two cases (1.7%) of primary lesions originating in the orbit (one of which was a uveal melanoma), two cases (1.7%) originating in the chest and cases (6.9%) starting in the anal canal and another case in the urethra. The mean age was 54 years, and the dimensions ranged from 0.3 to 5 cm in maximum diameter. In almost 90% of the cases the immunohistochemistry described positivity for S-100 protein and HMB-45, with 7% of the cases positive for Neuron-Specific Enolase (NSE) and 23.5% of the lesions expressed the carcinoembryonic antigen.

In the vast majority of cases, the clinical suspicion was that of an atypical pigmented lesion, suggestive of malignant melanoma. In a small number of cases, amelanotic lesions were appreciated.

## 4. Discussion

Malignant melanoma continues to represent a very frequent malignant neoplasm, rapidly increasing worldwide, and this increase is occurring at a faster rate than that of any other cancer except lung cancer in women [1,6]. Histopathological diagnosis is still the gold standard for programming subsequent steps in the therapeutic diagnostic path of the affected patient [6], and a correct morphological and immunohistochemical recognition is the basis for improving the outcome of patients (in fact, the five-year relative survival rate for patients with stage 0 melanoma is 97%, compared with about 10% for those with stage IV disease) [1,2,3,4,5]. Among the best known different histological patterns, there are unusual and bizarre forms of MM [6] whose knowledge is important to reduce and avoid the risk of wrong diagnoses. In this view, BCM represents a very rare variant (<1% of all histological forms of melanoma), defined by at least the presence of 50% of melanocytes with balloniform histological appearance [7]. Over the years, there have been different reports of BCM since Gardner’s first report in 1970 [9], which reported a case of BCM developed at the level of the back of an older patient. Since then, descriptions of this entity have multiplied [10,11,12,13,14,15,16,17,18,19,20,21,22,23,24,25,26,27,28,29,30,31,32,33,34,35,36,37,38,39,40,41,42,43,44,45,46,47,48,49,50,51,52,53,54,55,56,57,58,59,60,61,62,63,64,65,66,67,68,69,70,71,72,73,74,75,76,77,78]; there are up to about 115 patients described in the literature, according to our review conducted and presented in this paper. From the analysis of the studies included, the most represented primitive localizations turned out to be the back, the lower and upper extremities, the choroid and the district head/neck, with also two rare cases to depart from the conjunctiva. This heterogeneity of distribution with predominance of the back has been found also in our new four described cases (two cases to the back, one left leg case and one right flank case). As described in the literature, even in our presented cases, there were no distinctive clinical characteristics, being generally present the suspicion of MM. In this regard, in recent years, some authors [57] have tried to look for suggestive and distinctive dermaoscopic criteria for the diagnosis of BCM. Resente F. et al., for example, have found that elements such as yellowish structureless areas, white lines, irregular hairpin-shaped and curved vessels can be suggestive of BCM. Regarding the prognosis, from the analyzed works it does not seem that there is a substantial difference compared to the conventional melanoma, always depending on the thickness of Breslow; therefore, the degree and depth of balloon cell changes do not affect the prognosis.

An aspect of great importance for the dermatopathologist is represented by the differential diagnostics with benign and/or malignant lesions to balloniform cells (such as the nevus, a balloon cell) or to other skin neoplasms to clear cells. The differential diagnosis between nevus and melanoma balloon cells can be very complex, as both of these entities may present very similarly [6,39,45]: in this regard, it may be necessary to dissect the sample extensively in search of areas of possible conventional malignant melanoma that may orient the diagnosis in the right direction. On the contrary, in the case of rather mild melanocytes, without cytological features being atypical, we can think of the diagnosis of nevus as balloon cells. Consideration should also be given to the possibility of being faced with a Spitz a balloon cell nevus [79,80,81] where the presence of certain histological details may help to orient oneself. In the case of large cells and epithelioids, with a ground glass cytoplasm and vesicular nucleus, in the absence of significant mitotic activity and with presence of epidermal hyperplasia, we can reasonably think of a Spitz balloon cell nevus, especially in the case of persons under 20 years of age [6,80]. BCM may also be confused with non-melanocytic entities, including a classic differential diagnosis of renal cell carcinoma, but also with lesions such as clear-cell sarcoma (malignant melanoma of soft parts), xanthoma, hibernoma and clear-cell carcinoma of the lung, ovary and endometrium. Regarding clear-cell melanoma, although some authors have proposed a distinction with BCM, we tend to avoid using this nosographic category, as it can be easily confused with clear-cell sarcoma [6]. We also remember entities such as clear-cell syringoma, granular cell tumor, malignant eccrine acrospiroma, sebaceous carcinoma, atypical fibroxanthoma and lepromatous leprosy. In all these cases, immunohistochemical investigations and essential integration with clinical-anamnestic information may help in the correct nosographic classification [41,42,43,44,45,46,47,48,49,50,51,52,53,54,55,56,57,58,59,60].

In recent years, some authors such as Chen Y. have described cases of BCM developing a mutation of BRAFV600E in the metastatic setting and, therefore, brought attention to how this entity, despite the peculiar morphological characteristics, is able to behave also from the molecular point of view as a conventional MM. This is already affecting the therapeutic side, as shown by recent papers [67].

## 5. Conclusions

In this work we have presented four new cases of BCM, and covering a rather long period of time, we ended up dwelling on the latest molecular acquisitions also in the context of such a rare variant of MM. We believe that future studies with numerous case series are essential not only to increase the knowledge of the pathophysiology of this neoplasm but also to correctly evaluate the response of BCM patients to new oncological therapies.

## Figures and Tables

**Table 1:** Clinical features of patients with balloon cell melanoma.

Number of Patient	Age	Gender	Localization	Clinical Appereance
1	76	F	left hand back	Malignant melanoma
2	75	M	back	Malignant melanoma
3	36	F	Left leg	Dysplastic nevus
4	51	M	Right side	Malignant melanoma

**Table 2:** Summary table of all cases searched in the literature and reported in this review.

Author(s)	Year	Number of Patient	Localization	Clinical Appeareance	Primitive/Metastastic
Gardner et al. [9]	1970	1	back	MM	primitive
Ranchod, M. [10]	1972	2	Right calf and inguinal lymph node	Metastasis of MM and soft tissue tumour	both metastatic
Riley, F.C [11]	1974	2	Ciliary body	Pigmented lesion	primitive
Rodrigues et al. [12]	1976	3	Choroid	Pigmented lesion	primitive
Gatteschi et al. [13]	1978	1	Back	MM	primitive and then metastatic
Jakobiec et al. [14]	1979	1	Ciliary body	Pigmented lesion	primitive
Søndergaard et al. [15]	1980	1	Back	MM	primitive
Ferracini et al. [16]	1982	1	Cerebellar	MM	metastatic
Friedman et al. [17]	1982	2	/	/	metastatic
Khalil et al. [18]	1983	1	Choroid	pigmented lesion	primitive
Horton et al. [19]	1983	1	arm	MM	primitive
Fievez et al. [20]	1984	1	back	MM	primitive
Peters et al. [21]	1985	1	Back with satellitosis	MM	primitive
Driot [22]	1986	1	Choroid	pigmented lesion	primitive
Da [23]	1987	7	anorectal	pigmented lesion	Primitive (7)
Driot et al. [24]	1987	1	Choroid	pigmented lesion	primitive
Aloi et al. [25]	1988	2	Back and arm	Pigmented lesion and amelanotic lesion	primitive (2)
Margo et al. [26]	1988	1	conjunctiva	pigmented macule	primitive
Napoli [27]	1988	1	arm	pigmented lesion	primitive
Heid [28]	1988	1	forearm	MM	primitive or metastatic ?
Akslen et al. [29]	1989	1	unknown	/	metastatic
Martinez et al. [30]	1990	1	eye	pigmented lesion	metastatic (liver)
Kao et al. [31]	1992	34	various site	pigmented and/or amelanotic lesion	primitive and then metastatic
Messmer et al. [32]	1992	1	uveal melanoma	pigmented macule	primitive
Cardesi et al. [33]	1993	1	lymph node	not detected	metastatic
Megahed et al. [34]	1994	1	/	MM	primitive (polipoid)
Mowat et al. [35]	1994	2	back (2)	MM (2)	primitive (2)
Adamek et al. [36]	1995	1	meninges	pigmented lesion	primitive from meningeal nevus
Kawamura et al. [37]	1995	1	forearm	MM	metastatic
Kiene et al. [38]	1996	1	back	MM	primitive
Gregel et al. [39]	1998	1	back	MM	primitive
Terayama et al. [40]	1999	1	arm	/	primitive
Requena et al. [41]	2001	1	back	MM	primitive and then metastatic
August et al. [42]	2001	1	unknow	/	metastatic
Baehner et al. [43]	2005	1	unknow	laterocervical swelling (right)	metastatic
Hoque et al. [44]	2005	3	Back (2) and arm (1)	MM (3)	primitive (3)
McGowan et al. [45]	2006	1	back	pigmented lesion	primitive
Plaza et al. [46]	2010	2 of 192 lesions	back	MM	primitive
Lee et al. [47]	2011	1	neck	neck swelling	primitive and then metastatic
Gessi et al. [48]	2011	1	brain	MM (skin)	primitive, then metastatic
Richardson et al. [49]	2012	1	cerebellum	MM (skin)	primitive, then metastatic
Inskip et al. [50]	2013	1	back	pigmented lesion	primitive
Bal et al. [51]	2013	1	anal canal	pigmented macule	primitive
Maher et al. [52]	2014	1	left forearm	pale nodule	primitive
Bures et al. [53]	2015	1	right tibia	amelanotic lesion	Metastatic from head BCM
Han et al. [54]	2014	1	right shin	Black nodule	primitive
Duman et al. [55]	2014	1	chest	papule	Primitive with satellitosis
McComiskey et al. [56]	2015	1	urethra	nodule	Primitive urethral MM
Seabra Resende et al. [57]	2019	1	right leg	reddish nodule	primitive
Inskip et al. [58]	2016	1	right posterior upper arm	atypical pigmented lesion	primitive
Hattori et al. [59]	2016	1	left lumbar region	atypical lesion	Primitive and then metastatic
Chavez-Alvarez et al. [60]	2017	1	chest	pigmented lesion	primitive
Iliadis et al. [61]	2017	1	unknown	/	metastatic to temporal lobe
Saharti et al. [62]	2017	1	left scapula	hyperpigmented lesion	primitive and then metastatic
Friedman et al. [63]	2018	2	back (1) left shoulder (1)	pigmented papule (1) black macule (1)	primitive (2)
Farah et al. [64]	2018	1	lymph node swelling	previous MM on feet	metastatic
Ravaioli et al. [65]	2018	1	chest	reddish nodule	primitive
Caltabiano et al. [66]	2019	1	back	amelanotic papule	primitive
Goto et al. [67]	2019	1	brain	nodule	metastatic
Chen et al. [68]	2021	1	upper conjunctiva	brownish noduloplauques	metastatic
Wei et al. [69]	2021	1	left infraorbital fold	subcutaneous nodule	primitive
García-Piqueras et al. [70]	2021	3	back (2) and arm (1)	pigmented lesion	primitive (3)
Laforga et al. [71]	2021	1	neck	pigmented lesion	metastatic to the parotid

Legend: MM: malignant melanoma.

## Data Availability

Not applicable.
